# Vibrating Exercise Equipment in Middle-Age and Older Women with Chronic Low Back Pain and Effects on Bioelectrical Activity, Range of Motion and Pain Intensity: A Randomized, Single-Blinded Sham Intervention Study

**DOI:** 10.3390/biology11020268

**Published:** 2022-02-08

**Authors:** Grzegorz Zurek, Martyna Kasper-Jędrzejewska, Iwona Dobrowolska, Agata Mroczek, Gerda Delaunay, Kuba Ptaszkowski, Tomasz Halski

**Affiliations:** 1 Department of Biostructure, University School of Physical Education, I.J. Paderewskiego 35, 51-612 Wroclaw, Poland; grzegorz.zurek@awf.wroc.pl (G.Z.); gerda.delaunay@awf.wroc.pl (G.D.); 2 Institute of Health Sciences, University of Opole, Katowicka 68, 45-060 Opole, Poland; iwona.dobrowolska@uni.opole.pl (I.D.); agata.mroczek@uni.opole.pl (A.M.); tomasz.halski@uni.opole.pl (T.H.); 3 Department of Clinical Biomechanics and Physiotherapy in Motor System Disorders, Faculty of Health Science, Wroclaw Medical University, Grunwaldzka 2, 50-355 Wroclaw, Poland; kuba.ptaszkowski@umed.wroc.pl

**Keywords:** vibrating exercise equipment, women, chronic low back pain, surface electromyography

## Abstract

**Simple Summary:**

Physical activity is often recommended as part of the management of chronic low back pain, which is one of the most common musculoskeletal disorders. Vibrating exercise equipment is used despite little scientific evidence to support its effectiveness in the prevention and treatment of musculoskeletal problems. The aim of this study was to evaluate the efficiency of using vibrating exercise equipment in women with chronic low back pain. Here, 92 women aged 49–80 years were assigned to one of two groups: the experimental and the control group. The intervention consisted of aerobic exercises with specific handheld equipment. Both groups performed physical activity twice weekly for 10 weeks. The erector spinae muscles’ bioelectrical activity, the lumbar range of motion and pain intensity were measured in all participants at baseline and after 10 weeks. Compared with baseline measures, there was a significant decrease in the bioelectrical activity of the erector spinae muscles during flexion movement, rest at maximum flexion, extension movement and rest in a prone position; an increase in the lumbar range of motion and a decrease in pain intensity following a program of physical activity with vibrating exercise equipment. No significant changes were found in intergroup comparisons; however, physical activity with vibrating exercise equipment could be a prospective strategy for increasing lumbar range of motion and decreasing pain and erector spinae muscle activity in people with chronic low back pain.

**Abstract:**

**Background:** Chronic low back pain (CLBP) is one of the most common musculoskeletal disorders. Physical activity (PA) is often recommended as part of the management of CLBP, but to date, no one particular exercise has been shown to be superior. Vibrating exercise equipment (VEE) is widely available and used despite little scientific evidence to support its effectiveness in the prevention and treatment of musculoskeletal problems. The aim of this study was to evaluate the efficiency of using VEE compared with sham-VEE in women with CLBP. **Methods:** A randomized (1:1 randomization scheme) single-blinded sham-controlled intervention study was conducted. Through simple randomization, 92 women aged 49–80 years were assigned to one of two groups: VEE (the experimental group) and sham-VEE (the control group). The VEE and sham-VEE intervention consisted of aerobic exercises with specific handheld equipment. Both groups performed physical activity twice weekly for 10 weeks. The erector spinae muscles’ bioelectrical activity (using an eight-channel electromyograph MyoSystem 1400L), lumbar range of motion (Schober’s test) and pain intensity (visual analog scale) were measured in all participants at baseline and after 10 weeks. **Results**: There was a significant decrease in the bioelectrical activity of the erector spinae muscles during flexion movement (left: Me = 18.2 before; Me = 14.1 after; *p* = 0.045; right: Me = 15.4 before; Me = 12.6 after; *p* = 0.010), rest at maximum flexion (left: Me = 18.1 before; Me = 12.5 after; *p* = 0.038), extension movement (right: Me = 21.8 before; Me = 20.2 after; *p* = 0.031) and rest in a prone position (right: Me = 3.5 before; Me = 3.2 after; 0.049); an increase in lumbar range of motion (Me = 17.0 before; Me = 18.0 after; *p* = 0.0017) and a decrease in pain intensity (Me = 4.0 before; Me = 1.0 after; *p* = 0.001) following a program of PA in the VEE group. **Conclusions:** No significant changes were found in intergroup comparisons. The beneficial changes regarding decreased subjective pain sensation in the VEE and sham-VEE groups may be due to participation in systematic physical activity. However, PA with vibrating exercise equipment could be a prospective strategy for increasing lumbar range of motion and for decreasing pain and erector spinae muscle activity in people with CLBP.

## 1. Introduction

Chronic low back pain (CLBP) is one of the most common health complaints in adults [1]. CLBP affects independence, the mental state and physical activity (PA) [2,3]. Conservative treatment of CLBP often involves PA in the form of specific training sessions [4,5] or exercises [6]. To date, there are no clear guidelines to suggest the superiority of any one exercise method [7]. A 2017 review by Geneen et al. [8] found that there is poor scientific evidence to support the use of PA in reducing pain in the general population, and the authors suggested the need for continuing research into new solutions for conservative treatment of chronic pain [8]. Other studies have found that regular PA in the older adults alleviates anxiety and depression, and improves cognitive function while also enhancing mobility, balance and upper limb function [9]. Furthermore, PA also prevents the formation of fibrosis in muscle tissue and has a role to play in promoting the anti-inflammatory properties of macrophages, reducing tissue sensitivity and metabolism. Use of PA also has a positive impact on myofascial tissue and supports the concept of “physical activity as medicine” [10]. Mora and Valencia [11] believe that for PA to have optimum health benefits in older adults, it must consist of aerobic, strength, flexibility and proprioceptive exercises. Moreover, the PA must be tailored to take past injuries, hip and knee function, chronic disease, prescribed medications and nutrition into account [11]. The use of vibration stimuli in medicine and rehabilitation is becoming increasingly popular [12]. Vibration provides a strong proprioceptive stimulus [13] which can improve body balance and gait re-education in older adults [14,15], and can reduce pain by stimulating the afferent alpha and beta motoneurons and inhibiting nociceptive pain fibers [16]. Vibration stimuli have been shown to lead to improvements in muscle strength [17] and muscle mass [18,19], a reduction in obesity [20], and an improvement of gait in neurological patients [21,22]. Moreover, vibration can be applied directly (local vibration) to the muscle [17,23] or indirectly using vibrating platforms (e.g., whole-body vibration, WBV). The use of WBV in conjunction with exercise has been studied in randomized clinical trials [24,25,26], but thus far, no comprehensive review evaluating specific interventions and protocols, especially in the case of older adults has appeared in the Cochrane Library. A review published in 2019 by Leite et al. [27] suggested there is not enough evidence to support the use of WBV in clinical practice among people with various disabilities [27]. Furthermore, no standard clinical guidelines are available for WBV physical activity in relation to curing CLBP [28]. There also may be a role for exercise using vibrating exercise equipment (VEE) as an alternative to exercises on a WBV platform [29,30,31]. VEE applies local vibration from the hand to the rest of the body. The frequency produced by VEE was measured at the soft grip on the top of the rings during movement. It ranged from 0 to about 460 Hertz and showed a mean amplitude at about 60 Hertz (information provided by the manufacturer). The frequency is not dependent on any electronic motor or device but instead is created by natural and dynamic movements created by the swinging of the rings in different directions; therefore, it does not achieve a constant frequency. The movement always starts at 0 Hertz (the resting position) and reaches its highest frequency at the midpoint of the movement until changing direction to the resting point again [29,30]. However, little is known about the efficacy of combining this specific exercise equipment with vibration to enhance low back pain improvements. We identified two studies on the use of VEE in people with cancer. Crevenna et al. [30] assessed the quality of life and the strength of the upper limb muscles in women with breast cancer before and after a 12-month intervention in the form of physical activity with VEE. It was found that exercising with the VEE was safe and improved the quality of life and upper limb muscle strength. In a pilot intervention study, Cenik et al. [29] used the same device in women with breast cancer and evaluated participants’ quality of life, upper limb muscle strength, body composition and 6-min walk test. After 3 months of physical activity with the vibration-generating device, there was an improvement in all the measured parameters. These projects are not directly comparable with our study; however, they provide evidence that the equipment used in our project is safe and well tolerated. Both of the aforementioned studies showed encouraging results and their authors recommended further research on the effectiveness of VEE.

Recent World Health Organization data have confirmed the rapid aging of the population around the world [32]. In Poland, the long-term senior policy for 2014–2020 encouraged the creation of physical activity programs as part of the so-called concept of healthy aging. These programs aim to be diverse and innovative, and function to encourage older people to participate in various forms of physical activity. So far, to our knowledge, the bioelectrical activity of the erector spinae muscles, lumbar range of motion (ROM) and reported low back pain in middle-aged and older women has not been studied in conjunction with exercises using vibrating equipment. Further research is needed into the benefits of a combined exercise intervention program involving muscular strength, flexibility and aerobic fitness for CLBP patients, as the literature has supported the use of each of these fitness areas individually, but more research should be conducted combining all three [33]. Despite encouraging evidence on the usefulness of exercise involving vibration in people with CLBP, the practical application of such research findings in this area remains limited. Programs and exercises utilizing vibration equipment are typically not well reported or not reported at all. 

Wilke et al. [34] have carried out a systematic review in order to find evidence of myofascial continuity between the trunk and upper limb muscles. The analysis revealed the presence of three myofascial connectivities between the trunk and the upper limbs: the dorsal arm chain, the ventral arm chain and the lateral arm chain. Two myofascial continuities start on the dorsal arm chain: both latissimus dorsi muscles and the infraspinatus and teres minor muscles fuse with the triceps brachii. At the elbow, the triceps brachii muscle merges with the small anconeus muscle, which then connects to the extensor carpi ulnaris of the lower arm [34]. It seems that chronic low back pain and methods of their treatment, due to fascial connections with other regions of the body [35], should be considered from the aspect of the body as a whole, in agreement with the biotensegrity biomechanical theory [36,37]. The evidence supports this theory, pointing toward functional and clinically relevant myofascial continuity [38,39]. Several authors [28,40,41,42] have shown that adding WBV to specific exercise could increase muscle activation of the lumbar–abdominal muscles in young patients with CLBP. According to VEE, during the swinging movements of the arms, the four steel balls inside the tube not only create vibration but also momentum due to the acceleration of the VEE and the inertia of the steel balls. In the resting position, the VEE weighs about 0.5 kg, which can change to about 5 kg through a dynamic swinging movement. We conclude that the momentum and vibration created by the swinging movement could affect the lumbar–abdominal muscles, which are important factors for CLBP. Therefore, the main goal of this study was to compare the ES muscles’ bioelectrical activity, lumbar ROM and low back pain in middle-aged and older women participating in a 10-week physical activity program with VEE and sham-VEE. In addition, the correlations among these parameters was evaluated. The main hypothesis was that the intervention of PA with VEE would decrease the resting and functional bioelectrical activity of the erector spinae muscles, increase the lumbar ROM and reduce low back pain. The present study has clinical relevance. If there were intergroup differences, then the simple handheld swinging-ring system could be used by physiotherapists in individuals with CLBP. If it were possible to prove the beneficial influence of the local vibration generated by the handheld swinging-ring system in people with CLBP, the device could be used in the work of a physiotherapist. This would be both economically important (reducing labor costs) and easy to use by the patient, as he or she would be able to do the exercises themselves.

## 2. Materials and Methods

### 2.1. Study Methodology

A randomized intervention study assessed the resting and functional bioelectrical activity of the erector spinae muscles before and after a 10-week PA program using VEE (experimental group). A control group was used, which performed the same 10-week PA program, but with sham non-vibrating equipment (sham-VEE). The project was carried out between September 2016 and June 2017, and was based on the Consolidated Standards of Reporting Trials (CONSORT) guidelines. The study was approved by the Bioethical Committee at Opole Medical School in Poland (24 October 2016, No. 44/2016) and registered on the Australian New Zealand Clinical Trials Registry platform (1 December 2016, number ACTRN12616001661460). The study was carried out in the Functional Research Laboratory of the Physiotherapy Department and used the gym of the Opole Medical School, Poland.

### 2.2. Study Participants

The target group included women between 50 and 80 years of age recruited from the University of the Third Age of Health and Beauty of Seniors of the Opole Medical School, Poland. Written informed consent was obtained from all participants included. The inclusion criteria were: aged over 49, a history of bilateral chronic pain in the lumbar spine lasting >6 months (they had completed the standard care procedure for acute LBP) and provision of signed voluntary consent to participate in the project. Exclusion criteria were: age <50 years old, acute pain in the lumbar spine requiring pharmacological treatment lasting <6 months, lack of pain in the lumbar spine, unstable hypertension, an inability to perform standing exercises, hip endoprosthesis and a lack of voluntary consent to participate in the project. Based on the criteria, 92 women were included in the project. The estimated sample size was calculated on the basis of the pilot study. The means and standard deviations of lumbar ROM before and after the intervention were used in the analysis of estimating the sample size. On the basis of the parameters, an estimated sample size equal to 39 participants in each group was obtained. In addition, the risk of losing patients in the follow-up assessment (10%) was assumed. The final sample size equaled a minimum of 43 participants in each group. The estimation of the sample size was performed using Statistica 13 (TIBCO Software Inc.). Participants were randomly assigned to the experimental group (VEE group, n = 43) or the control group (sham-VEE group, n = 49). Randomization was carried out using computer-generated random numbers (simple randomization). The participants were randomly assigned to the groups in a 1:1 ratio. Both groups were subjected to a systematic PA intervention (a 60-min exercise session twice a week for 10 weeks) with the use of equipment. The equipment used in the experimental group was a handheld device which generated vibrations and momentum when it was set in motion. The same equipment was used in the control group, but it did not generate vibration and momentum during movement (sham-VEE). Both pieces of equipment were identical in weight and external structure. The participants were not aware which intervention they were receiving (single-blinded).

### 2.3. Methods

The bioelectrical activity of the right and left erector spinae muscles in the lumbar spine of each participant was measured using an eight-channel electromyograph (MyoSystem 1400 L, Noraxon, Scottsdale, AZ, USA), MyoResearch software (Noraxon) and compatible, disposable, self-adhesive Ag/AgCl electrodes. The participants assumed a comfortable, casual position lying face down. The study was conducted in accordance with SENIAM guidelines [43].

The electromyographic (surface electromyography, sEMG) signals were subjected to standard post hoc processing with rectification (purification) and smoothing using an RMS calculation algorithm. The sEMG recording frequency was set to the range of 10 to 450 Hz with a high-pass filter cutoff of 10 Hz and a low-pass filter cutoff of 500 Hz. The level of common-mode rejection was a minimum of 100 dB, and the input impedance for the sEMG channels was higher than 100 mΩ. The system had high sensitivity in terms of recording sEMG signals (1 µV). The erector spinae (ES) muscles’ bioelectrical activity was measured during “rest” (45 s of ES activity at rest before any functional measurements), and at “functional tone” (measurement in the standing position lasting 45 s, during which the patient performed lumbar flexion, with maintenance of rest at maximal flexion and extension). Normalization to maximal or submaximal contractions has not been considered a solution for low back pain patients, and thus, non-normalized EMG amplitudes were preferred in our study [44].

The original Schober’s test was used to measure the lumbar ROM. The assessment was performed at the level of L5, with 2 points marked 5 cm below and 10 cm above L5. The participants were then asked to perform a forward bend, flexing the torso and touching their toes if possible while keeping the knees straight. The distance between the 2 points was measured in this position [45]. 

Participants were asked to rate the intensity of pain on a visual analog scale (VAS). Scores were measured by asking each participant to mark the intensity of pain on a 10 cm line, annotated with “no pain” at one end and “as bad as it could possibly be” at the other end [46].

### 2.4. Intervention

The PA with VEE and sham-VEE is detailed in Table A2 (Appendix A). These were overseen by physiotherapists trained in using vibrating equipment. The VEE is a handheld spiral tube with 4 steel balls within it. Movement in the sagittal, frontal or horizontal plane (e.g., a swinging motion) sets the metal balls in motion. The movement of the balls creates a vibration of 60 Hz, which is transmitted through the handles of the tool. The weight of the static equipment is 0.5 kg; however, it can reach up to 5 kg during movement due to centrifugal forces. 

**Figure A1:**
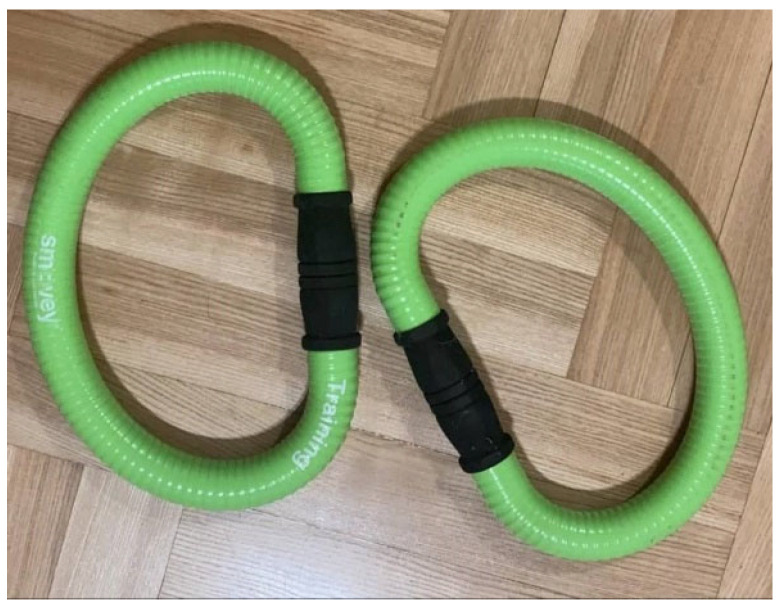
Smovey rings.

The handheld vibration exercise equipment used in the study consisted of 4 steel balls (26 g each, diameter = 24 mm) located inside a spiraled tube made of soft (65%) and hard (35%) PVC (internal groove protruding, helix pitch 6.2 mm) and a grip with cushioning elements. The device name “Smovey” comes from the three words: “swing”, “move” and “smiley” (Appendix A, Figure A1).

Each participant was instructed how to hold the equipment properly and how to perform the correct swinging movement in different planes whilst maintaining proper body posture. The PA comprised simple movements of moderate intensity. The level of the intensity (perceived exertion) was based on the subjective assessment of each participant expressed during the exercise using the Borg scale. Perceived exertion was measured by the Borg RPE scale, which contains both verbal anchors and a numerical scale. The numbers range from 6 to 20, while the verbal anchors start at 6, which is labeled as “least effort”, 7 is “very, very light”, 9 is “very light”, 11 is “fairly light”, 13 is “somewhat hard”, 15 is “hard”, 17 is “very hard”, 19 is “very, very hard” and 20 is “maximum effort”. After each set of exercises, the physiotherapist asked the participants to rate her level of effort in performing the exercises on the Borg scale (“How hard you feel your body has worked?”) [47]. Participants performed the PA regime for ~60 min twice per week for a total duration of 10 weeks. The exercises were performed in the same order for both groups. Each participant had their own exercise mat and set of exercise equipment for each session. Each exercise was demonstrated by a physiotherapist, and the participants were asked to copy each movement. Participants were reminded at each session of the correct starting position and tonicity, the importance of keeping slightly bent knee joints, the correct way to hold the equipment (keeping the wrists stiff) and the correct range of arm movement. The classes were always held at the same time. Participants were dressed in loose sports clothing and footwear that did not constrict movement. The demonstrator set the pace of the exercises.

### 2.5. Statistical Analysis

Statistica 13 software was used to perform the statistical analysis (StatSoft, Inc., Tulsa, OK, USA). Due to the lack of a normal distribution in the obtained results, the medians, quartiles (Q1, Q3) and range of variability were calculated for each measurable variable. The frequency of occurrence (percent) was calculated for qualitative variables. All the tested quantitative variables were checked by means of the Shapiro–Wilk test to determine the type of distribution. Intragroup comparisons (before vs. after the intervention) were performed using the Wilcoxon test. The differences between the results obtained in the experimental group and the control group were determined using the non-parametric Mann–Whitney U-test. For all comparisons, a level of α = 0.05 was assumed. Correlations between quantitative variables were analyzed using the Spearman correlation coefficient.

## 3. Results

**Figure 1:**
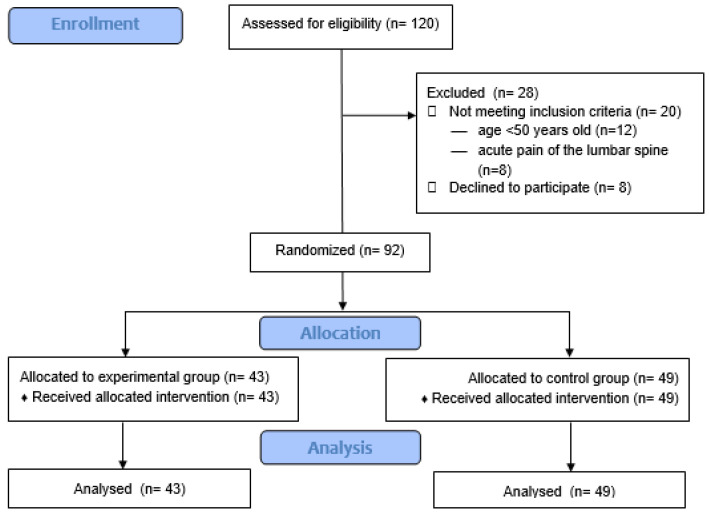
Flow chart of study enrollment, allocation and analysis.

In total, 120 participants were enrolled in the study. Based on the inclusion and exclusion criteria, 92 women were included in the project. They were randomly assigned to one of the two groups: the experimental group (n = 43) or the control group (n = 49). All 92 subjects completed the 10-week exercise program (Figure 1) following the intention to treat principle.

### 3.1. Characteristics of the Participants

Table 1 shows the characteristics of the experimental group and the control group. The age in the experimental group ranged from 50 to 76 (mean = 66.0 years old) and that in the control group ranged from 56 to 80 (mean = 66.0 years old). There were no statistical differences in the characteristics of the two groups.

### 3.2. Erector Spinae sEMG Results

A comparison of the results of the sEMG measurements is presented in Table 2. Compared with the baseline measures, there was a statistically significant decrease in the flexion sEMG of the left and right ES muscles in the VEE group following the PA intervention. Moreover, there was a decrease in the sEMG activity of rest at maximal flexion in the left ES muscle and in the sEMG activity of extension of the right ES observed in the VEE group. The sEMG of the right ES muscle in the resting position also decreased significantly following the intervention. There were no statistically significant changes in sEMG following the PA intervention observed in the sham-VEE group. Moreover, there were no statistically significant differences in the change in sEMG between the VEE and sham-VEE groups.

### 3.3. Lumbar ROM Results 

The measurements of lumbar ROM are presented in Table 3. Compared with the baseline measures, there was a statistically significant improvement in the ROM following the PA intervention in the VEE group. There were no statistically significant changes in ROM following the PA intervention observed in the sham-VEE group. There were no statistically significant differences observed between the VEE and sham-VEE groups.

### 3.4. Pain Scores (VAS)

Both the VEE and sham-VEE groups demonstrated a decrease in pain intensity following the intervention, which is shown in Table 4. There was a statistically significant decrease in VAS in the VEE group, from 4 to 1 following the exercise intervention. Similarly, the level of pain expressed on the VAS scale in the sham-VEE group decreased from 5 to 1 after the end of the study, which was also statistically significant. There was no statistical difference in VAS score between the groups either before or after the intervention.

## 4. Discussion

The aim of the study was to compare the effect of a 10-week exercise program, using either VEE or sham-VEE, on the bioelectrical activity of the erector spinae muscles in the lumbar spine, lumbar range of motion and reported pain in middle-aged and older women with chronic low back pain. The study also investigated whether bioelectric activity correlated with the ROM of the lumbar spine and reported subjective pain levels. It was hypothesized that after the end of the 10-week PA intervention with VEE, the bioelectrical activity of the erector spinae muscles would be significantly lower compared with their activity before the intervention, particularly in the experimental group and in comparisons between the groups. It was also hypothesized that a reduction in the measured bioelectrical activity of the erector spinae muscles would correlate with increased lumbar ROM and reduced pain intensity. The results showed a significant reduction in the bioelectrical activity of the erector spinae muscles following the PA intervention in the experimental group; however, there was no statistical difference when comparing the experimental group with the control group. Moreover, there was no significant correlations between the measured bioelectrical activity of the erector spinae muscles and ROM. To our knowledge, the study was the first randomized sham trial that has been conducted to evaluate the effects of exercise with VEE on these parameters. The study aimed to answer the question of whether PA with VEE is more effective than PA with sham-VEE in middle-aged and older women suffering from CLBP.

### 4.1. Erector Spinae sEMG

It appears that the reduction in the bioelectrical activity of the ES muscles in our study after the 10-week intervention in the experimental group is a positive phenomenon. This would suggest that the use of VEE has a beneficial stimulatory effect on the nervous system responsible for ES muscle innervation, as well as possibly improving the blood supply to the muscle due to changes in its tone. However, measurements of bioelectrical activity of the ES muscles in patients with CLBP are still inconsistent [48,49]. Lima et al. [44] found that those suffering from CLBP had increased muscle activity, which was possibly caused by excessive stimulation of the nervous system. Participants with CLBP showed an increase in back muscle activity compared with asymptomatic participants, regardless of the type of functional task [44]; moreover, increased trunk muscle activity has been shown to be a key feature in the presence of pain [50]. Therefore, the reduction in resting and functional sEMG as demonstrated in our study may have resulted in a decrease in ES muscle hyperactivity, which is one of the factors contributing to pain onset and persistence in study participants. This study hypothesized that the use of VEE during PA would modify the level of bioelectric activity of the ES through myofascial connections between the upper and lower back [51]. The ES muscles are located between the lamina superficialis and the lamina profunda of the thoracolumbar fascia, which functions to carry mechanical loads [52] along with the back extensors and gluteal muscles [53]. The thoracolumbar fascia also contributes to movement coordination, stability and proprioception, and aids in promoting sliding and reducing friction during movement. Any trauma or pain may alter the sliding mechanism within the fascial plane [54]. Therefore, if fascial stiffness is increased, the nociceptors could be sensitized, causing the underlying muscles to be stiffer [55]. The studies by Nowotny et al., (2018) and Daneau et al., (2019) suggested that the reduction in the endurance and strength of the paraspinal muscles, which results in development of pain in this region, may be related to an increased number of Type II muscle fibers in patients with LBP pain compared with people who did not report pain in this region [56,57]. 

A review of literature showed that this type of vibration (particularly related to the VEE) and its use in exercise has not been extensively studied; therefore, it was difficult to compare our study with any other literature. A supplementary stimulus was also generated by the noise of the four steel balls rolling in the spiral tube, which generated an auditory feedback corresponding to the intensity of the vibration stimuli. Goossens et al. [13] conducted an experiment in which local vibration from 20 to 60 Hz applied to the triceps surae and back muscles was chosen as the “stimulation condition” to control the simultaneous activation of vibrotactile skin receptors. This study used magnetic resonance imaging (MRI) to evaluate brain activity when the local vibrating stimulus was applied to the erector spinae muscles in people with and without non-specific low back pain (NSLBP). The results indicated that patients with NSLBP were more cautious of movement and needed more time to complete tasks compared with the control group. In addition, MRI of patients with NSLBP showed activation of the right S2 cortex and the right primary auditory cortex (Heschl′s gyri), areas that are important for proprioception, during stimulation of the erector spinae muscles. Although there were no significant differences in the processing of proprioceptive information between participants with NSLBP and healthy participants, correlations of brain behavior showed that in order to maintain optimal proprioception to respond to postural changes, patients with NSLBP may experience increased activation of the regions responsible for sensory processing. The use of vibration stimuli also triggered increased activation of brain areas involved in threat detection and fear processing in some participants, which was associated with poorer proprioceptive posture control [13]. The results of our study indicated no statistically significant differences between the VEE and sham-VEE groups, which may suggest that the vibration generated by the equipment used did not affect the erector spinae muscles in the aspects of neurophysiology studied. 

### 4.2. Lumbar ROM

The reduction in ROM that occurs with age plays an important role in physical function [58]. Some authors suggested that people with CLBP are highly fearful and not only guard themselves during flexion and extension movements, but they also fear that pain will significantly limit their ROM, for example, during flexion–extension movements [59]. In this study, there was a statistically significant improvement in lumbar ROM after the PA intervention in the experimental group. The increase in ROM associated with the reduction in pain intensity within the painful lumbar spine may be associated with a restoration of soft tissue architecture in this region of the body and a potential reduction in the number of Type II muscle fibers [56,57]. According to Langevin et al., (2011), a potential consequence of chronic low back pain is fibrosis and adhesions that may inhibit independent motion of the adjacent connective tissue layers, which can restrict movement [60]. Since mobility range is closely related to soft tissue flexibility and because of the fact that mobility range decreases not only due to pain but also age [61], any increase in flexibility will be of particular value to middle-aged and older adults, as those who maintain better flexibility are more likely to be independent when performing functional everyday activities [61].

### 4.3. Pain Scores (VAS) 

This study showed a significant reduction in subjective pain sensations within the lumbar spine in both the experimental and the control group. If we assume that pain increases ES muscles’ bioelectrical activity, which, in turn, further increases pain, then reducing this subjective perception of pain may be the key element in breaking the vicious cycle of CLBP [62]. There are various theories regarding the impact of pain on the bioelectrical activity of muscles [63]. The results of research conducted by Arendt-Nielsen et al. [64] showed that there was no correlation between VAS reduction and a decrease in the erector spinae muscles’ bioelectrical activity. Our results showed some discrepancy between objective measurements of the bioelectric activity of the ES and subjective measurement of pain sensation. The change in the bioelectrical activity of the ES in the VEE group was simultaneously supported by a significant reduction in pain; however, a reduction in pain without a significant change in the bioelectric activity of the ES muscles was also observed in the sham-VEE group. From the point of view of the patient’s quality of life, such a significant reduction in pain in both groups is obviously a beneficial phenomenon. The results of the study also did not reveal whether PA with VEE or with sham-VEE was superior. This may partly stem from the fact that the participants were properly educated on how to perform each exercise, particularly the importance of maintaining correct posture during the PA. Maintaining correct posture, emphasizing the lumbar lordosis, during PA makes it possible to keep the various intricate structures of the back and spine healthy [65]. PA with the equipment proposed in our intervention study focused on the activation of the deep torso muscles, targeting the restoration of control and coordination of these muscles with the view to progressing to more complex and functional tasks that integrate the activation of the deep torso muscles as a whole.

### 4.4. Clinical Implications

The study aimed to answer the question of whether PA with VEE is more effective than PA with sham-VEE in middle-aged and older women suffering from CLBP. A surprising finding was that there was no difference between the VEE and sham-VEE physical activity groups (intergroup comparison). Only the VEE group had significant decreases in the ES muscles’ bioelectrical activity and increases in lumbar ROM. Both groups had significant decreases in VAS. These findings do not support our hypothesis that the intervention of PA with VEE, more than PA with sham-VEE, would decrease the resting and functional bioelectrical activity of the erector spinae muscles, increase the lumbar ROM and reduce low back pain because it provides a vibration effect. Thus, at this phase of the study, we are unable to confirm that in middle-aged and older women with CLBP, the local vibration generated by a simple handheld swinging-ring system during a physical activity program is more effective compared with exercises in which it does not occur. For planning physical activity in middle-aged and older women with chronic low back pain, exercise with VEE can be included but, based on our results, the beneficial changes observed in this study may result from systematic physical activity, not from the equipment used (no statistical differences in intergroup comparisons). In the case that an individual possesses exercise equipment such as VEE, this can only enrich aerobic training and expand the spectrum of exercises that can be performed, especially when aerobic exercise is routinely recommended to improve physical function in aging individuals [66,67].

### 4.5. Study Limitations

There was no long-term follow-up of the participants to see if any benefit was sustained long-term. The physiotherapists involved in this project were not blinded as to whether the devices used were vibrating or non-vibrating. The VEE used in the project could have been described better in technical terms, focusing on how the vibrations are generated—the project was based only on the information available from the manufacturer. Future work could, however, seek to explore approaches to the normalization of sEMG data, accounting for factors such as activities of daily living and regular physical activity, training participants to control the deep muscles of the trunk to stabilize the spine during perturbations, different training methods and other training equipment or vibration applied to the lower extremities. Another weakness was that the vibration never reached a constant frequency, which might be required (or devices with a higher frequency), which could be tested in future studies. Moreover, the weakness was that there were no minimum pain score criteria for LBP (range from 1 to 10), and including middle-aged and older participants with low chronic back pain scores (1–3) may have adversely impacted the overall pain-related outcome measures. Future studies should include participants with greater chronic LBP than scores of 1 or 2.

## 5. Conclusions

Application of local vibration from the hand to the rest of the body did not result in significant changes in the bioelectric activity of the ES, lumbar ROM or pain intensity in intergroup comparisons. The beneficial changes observed in this study regarding decreased subjective pain sensation in the VEE and sham-VEE groups may be due to participation in systematic physical activity rather than the vibration-generating equipment used. Physical activity with VEE increased lumbar ROM and decreased pain and the erector spinae muscles’ activity in middle-aged and older women with chronic low back pain. In future, well-designed studies with a large sample size should be conducted to assess the possible effects of the manual swinging-ring system. This will allow further exploration and validation of the benefits of PA with VEE for this age group.

## Figures and Tables

**Table 1:** Characteristics of the participants.

	VEE (Experimental) Group n = 43					Sham-VEE (Control) Group n = 49					*p*-Value
	Me	Min	Max	Q1	Q3	Me	Min	Max	Q1	Q3	
Age (years)	66.0	50.0	76.0	63.0	69.0	66.0	56.0	80.0	64.0	69.0	0.68 *
Height (cm)	160.0	146.0	176.0	156.0	165.0	160.0	149.0	171.0	155.0	164.0	0.89 *
Weight (kg)	75.4	47.1	107.5	64.2	85.0	70.6	49.3	177.7	63.0	77.0	0.17 *
BMI (kg/m^2^)	28.4	19.1	42.3	25.2	32.5	27.7	19.3	42.7	25.8	29.2	0.24 *

* Mann–Whitney U-test; Me—median; Mi—minimum; Ma—maximum; Q1—lower quartile; Q3—upper quartile.

**Table 2:** Intragroup and intergroup comparisons of the changes in erector spinae sEMG measured before and after the PA intervention (µV).

	VEE (Experimental) Group										Sham-VEE (Control) Group										*p*-Value *	*p*-Value **	*p*-Value ***	*p*-Value ****
	Before					After					Before					After								
	Me	Min	Max	Q1	Q3	Me	Min	Max	Q1	Q3	Me	Min	Max	Q1	Q3	Me	Min	Max	Q1	Q3				
Flexion sEMG—left ES	18.2	7.6	57.3	12	19.9	14.1	4.5	49.9	8.8	18.5	14.7	8.4	33.4	11.7	17.6	13.7	7.8	28.1	10.9	17.5	0.045	0.34	0.07	0.70
Flexion sEMG—right ES	15.4	6.6	39.9	12	19.8	12.6	5.6	30	9.1	16.7	13.9	6.5	45.2	10.2	17.3	12.3	4.6	30.4	10	16	0.010	0.22	0.30	0.87
Rest (in maximum flexion)—left ES	18.1	3.1	40.6	12	20.2	12.5	2.8	53.3	8	17.5	14.7	3.5	27.9	11.4	18.9	12.2	3.5	30.8	9.2	16.8	0.038	0.10	0.28	0.73
Rest (in maximum flexion)—right ES	15.5	2.7	32.9	10.3	20.6	11.1	2.3	29.4	7.2	16.9	13	3.2	46.3	9.9	17.8	10.9	3.1	30	7.7	17	0.06	0.14	0.31	0.95
Extension—left ES	22.9	10.2	47.4	17.2	31.6	20.3	4.5	54.1	13.4	28.5	18.2	3.8	48.3	14.4	25.6	18.5	8.3	58.9	14.7	25.1	0.06	0.57	0.13	0.89
Extension—right ES	21.8	8.5	38.9	15.3	29.1	20.2	5.9	45.4	14.2	24.1	17.7	8.1	48.6	13.9	23.6	15	7.8	43.9	12.3	23.3	0.031	0.64	0.07	0.29
Rest—left erector spinae	5.7	2.1	19.4	4	7.1	5.3	2.3	18.1	4.4	7.1	5.7	2.3	20.1	4.6	8.3	5.3	2.4	16.1	4.3	7	0.78	0.16	0.34	0.88
Rest—right erector spinae	3.5	1.9	16.2	2.8	4.9	3.2	1.5	7.3	2.5	4.6	3.4	1.6	18.5	2.7	4.8	3.2	1.9	11	2.5	4.4	0.049	0.37	0.52	0.88

* Experimental group: before vs. after (Wilcoxon test); ** control group: before vs. after (Wilcoxon test); *** comparison of “before” results: experimental group vs. control group (Mann–Whitney U-test); **** comparison of “after” results: experimental group vs. control group (Mann–Whitney U-test); Me—median; Mi—minimum; Ma—maximum; Q1—quartile lower; Q3—quartile upper; left ES—left erector spinae, right ES—right erector spinae.

**Table 3:** Intragroup and intergroup comparisons of the changes in lumbar ROM measured by Schober’s test before and after the PA intervention (cm).

	VEE (Experimental) Group										Sham-VEE (Control) Group										*p*-Value *	*p*-Value **	*p*-Value ***	*p*-Value ****
	Before					After					Before					After								
	Me	Min	Max	Q1	Q3	Me	Min	Max	Q1	Q3	Me	Min	Max	Q1	Q3	Me	Min	Max	Q1	Q3				
Distance between points in a standing position (cm)	13.0	10.0	16.0	12.0	14.0	14.0	12.0	18.0	10.0	15.0	13.0	10.0	15.0	12.0	14.0	13.0	10.0	15.0	12.0	14.0	0.0015	0.11	0.79	0.68
Distance between points in a flexing position (cm)	17.0	13.0	21.0	15.5	19.0	18.0	15.0	23.0	17.0	19.5	17.0	13.0	22.0	16.0	19.0	18.0	14.0	22.0	16.0	19.0	0.0017	0.09	0.88	0.29

* Experimental group: before vs. after (Wilcoxon test); ** control group: before vs. after (Wilcoxon test); *** comparison of “before” results: experimental group vs. control group (Mann–Whitney U-test); **** comparison of “after” results: experimental group vs. control group (Mann–Whitney U-test); Me—median; Mi—minimum; Ma—maximum; Q1—lower quartile; Q3—upper quartile.

**Table 4:** Intragroup and intergroup comparisons of the changes in VAS scores before and after the PA intervention.

	VEE (Experimental) Group										Sham-VEE (Control) Group										*p*-Value *	*p*-Value **	*p*-Value ***	*p*-Value ****
	Before					After					Before					After								
	Me	Min	Max	Q1	Q3	Me	Min	Max	Q1	Q3	Me	Min	Max	Q1	Q3	Me	Min	Max	Q1	Q3				
VAS	4.0	1.0	10.0	2.0	5.0	1.0	0.0	8.0	0.0	4.0	5.0	1.0	10.0	3.0	8.0	1.0	0.0	9.0	0.0	5.0	0.001	<0.001	0.07	0.90

* Experimental group: before vs. after (Wilcoxon test); ** control group: before vs. after (Wilcoxon test); *** comparison of “before” results: experimental group vs. control group (Mann–Whitney U-test); ****comparison of “after” results: experimental group vs. control group (Mann–Whitney U-test); Me—median; Mi—minimum; Ma—maximum; Q1—lower quartile; Q3—upper quartile.

## Data Availability

The datasets used and/or analyzed during this study are available from the corresponding author on reasonable request.

## Appendix A

**Table A1:** Correlation between the bioelectric activity (µV) of the erector spinae muscles and the ROM of the lumbar spine in the experimental and control groups.

		Experimental Group				Control Group			
		Lumbar ROM Before		Lumbar ROM After		Lumbar ROM Before		Lumbar ROM After	
		r_s_	*p*-Value	r_s_	*p*-Value	r_s_	*p*-Value	r_s_	*p*-Value
Before	Baseline	0.239019	0.102	0.123192	0.404	0.198600	0.2017	0.109459	0.4847
	Functional	0.106851	0.470	0.189222	0.198	−0.089447	0.5684	0.088912	0.1571
After	Baseline	0.086178	0.560	−0.057862	0.696	0.190182	0.2219	0.003511	0.9822
	Functional	0.005431	0.971	−0.008733	0.953	0.011753	0.9404	0.050751	0.7464
r_s_ = Spearman’s correlation coefficient									

**Table A2:** Overview of the exercise classes.

Course of the Class	Name and Description of Exercise	Time (min)
Welcome and introduction	The group is welcomed and the equipment is distributed. The participants are asked about their wellbeing and pain level, Participants are reminded of the principles of using the equipment, and the importance of maintaining correct and normal breathing during exercising.	5
Warm-up 5 min	Starting position (SP) = Participants stand with their arms by their sides. Shoulder circles are performed forward and backward.	1
	SP as above, with shoulder circles forward and backward, combined with a light stepping motion of the feet	1
	SP as above, marching the feet, swinging each alternate arm forward and backward.	1
	SP as above, marching the feet, with parallel arm movement forward and backward.	1
	SP with the arms by the sides holding the Smovey in each hand. The hands are moved inward and outward	1
Main part 32 min	SP as above, marching (four steps forward, four steps backward) with alternate arm movements and with lifting the knees up on every fourth step.	2
	SP as above, stepping out to the side with alternating abduction of each arm to the side.	2
	SP as above, raising each alternate leg upward, with alternating arm movement to the side.	2
	SP as above, with a step with raised knees, with movement of the arms up and down on the outside.	2
	SP as above, stepping with alternating arm movement front to back.	2
	SP as above, stepping with alternate arm movement forward and backward.	2
	SP as above, stepping forward and backward with weight transfer, with arms moving forward and backward, crossing at the front at the height of the chest.	2
	SP as above, diagonal movement of the legs forward and backward, with parallel shoulder movement sideways.	2
	SP as above, the Smovey joined together, held behind with both hands at the height of the hips, slight arm movement sideways.	2
	SP = standing in straddle position, arms at the height of the shoulders, crossing and abduction of the arms	2
	SP = lying on the side, arm movement above the head and then toward legs. Uppermost leg is lifted upward. Performed both right and left.	2
	SP as above, arms kept straight along the torso, the Smovey joined together, forward and backward movement of the arm and the leg in opposite directions. Performed right and left.	2
	SP as above, the knee of the upper leg is bent and moved forward, massage of the buttock with the Smovey. Performed right and left.	2
	SP = lying on the back, the Smovey is held behind the head with both hands, moving arms forward once to the right and once to the left of the torso.	2
	SP as above, left leg is bent at the knee, the heel is leaned against the ground, right leg lifted straight up and down with parallel arm movement toward the right leg. Repeated right and left.	2
	SP as above, legs are bent at an angle of 90°, the Smovey is held with both hands at the height of the chest. Moving legs sideways while moving arms in the opposite direction.	2
Cool down 5 min	SP standing with the Smovey held in each hand, bending forward with arms directed forward.	1
	SP = standing in straddle position, the Smovey is held with both hands, lifting the hands up and bending forward, moving the Smovey toward the floor.	1
	SP as above but bending to the sides.	1
	SP as above, the Smovey is held on the shoulders, with rocking sideways movements.	1
	SP as above, raising arms up while breathing in and lowering arms while breathing out.	1
Organizational matters	The end of the class, asking the participants about their perceived intensity of exercise using the Borg scale, saying goodbye.	3
